# Evolutionary Dynamics of Gene Expression During Thermal Adaptation in *Drosophila subobscura*

**DOI:** 10.1093/gbe/evag033

**Published:** 2026-03-24

**Authors:** Marta A Antunes, Marta A Santos, Mauro Santos, Margarida Matos, Pedro Simões

**Affiliations:** CE3C—Centre for Ecology, Evolution and Environmental Changes & CHANGE—Global Change and Sustainability Institute, Lisboa, Portugal; Departamento de Biologia, Faculdade de Ciências, Universidade de Lisboa, Lisboa, Portugal; CE3C—Centre for Ecology, Evolution and Environmental Changes & CHANGE—Global Change and Sustainability Institute, Lisboa, Portugal; Departamento de Biologia, Faculdade de Ciências, Universidade de Lisboa, Lisboa, Portugal; CE3C—Centre for Ecology, Evolution and Environmental Changes & CHANGE—Global Change and Sustainability Institute, Lisboa, Portugal; Departament de Genètica I de Microbiologia, Grup de Genòmica, Bioinformàtica I Biologia Evolutiva (GBBE), Universitat Autònoma de Barcelona, Bellaterra 080193, Spain; CE3C—Centre for Ecology, Evolution and Environmental Changes & CHANGE—Global Change and Sustainability Institute, Lisboa, Portugal; Departamento de Biologia, Faculdade de Ciências, Universidade de Lisboa, Lisboa, Portugal; CE3C—Centre for Ecology, Evolution and Environmental Changes & CHANGE—Global Change and Sustainability Institute, Lisboa, Portugal; Departamento de Biologia, Faculdade de Ciências, Universidade de Lisboa, Lisboa, Portugal

**Keywords:** evolutionary dynamics, gene expression, trajectories, warming, adaptation, experimental evolution

## Abstract

Global warming poses significant challenges to the persistence of biodiversity, requiring a deeper understanding of thermal adaptation, particularly at the genetic level. Examining gene expression changes offers critical insight into the molecular mechanisms driving adaptation, helping to identify which genes are activated or repressed during the adaptive dynamics. This study investigates the evolutionary dynamics of gene expression in *Drosophila subobscura* populations from different origins under progressive warming conditions. By analyzing transcriptomic changes across two generation gaps (after 9 and 23 generations of evolution), we found some complex evolutionary patterns of gene expression, including shifts from up to downregulation and vice versa, with the population of Dutch origin exhibiting greater variability in adaptive response than the population of Portuguese origin. Such complex dynamics involved enrichment of DNA replication—and DNA repair mechanisms in particular—in both sets of populations, although the timing and direction differed between them. We also found consistent downregulation of the immunity-related toll-NF-κB pathway in the replicated populations derived from the Dutch population. This study documents the dynamic and shifting nature of gene expression during adaptation to warming environments and highlights the critical role of population's history in shaping adaptive strategies. These findings deepen our understanding of the transcriptomic mechanisms driving thermal adaptation and provide a basis for predicting evolutionary responses to climate change.

Significance As global temperatures rise, understanding how organisms adapt at the molecular level is critical for predicting their survival. This study reveals that gene expression during thermal adaptation is not simply a matter of steady, directional change but instead follows dynamic and sometimes reversible paths—even in early generations. These findings challenge the assumption of directional adaptive trajectories and underscore the complexity of evolutionary responses to climate change.

## Introduction

In face of the escalating challenges posed by global warming (IPCC, 2022), understanding the adaptive capacity of populations is paramount to determine their persistence and the sustainability of ecosystems (Hoffmann and Sgrò 2011; Edelsparre et al. 2024). The loss of biodiversity, including declines in insect populations (Engelhardt et al. 2022), has raised questions about their ability to adapt to rising temperatures. However, adaptation is not simply a matter of whether populations can adapt, but how they adapt. Therefore, it is essential to understand the temporal dynamics and patterns of evolutionary change (Martin et al. 2023). To predict future responses to environmental challenges and estimate the rate at which populations can cope with novel conditions, it is essential to characterize evolutionary trajectories, rather than focusing only on evolutionary endpoints. Adaptation is a dynamic process, and understanding multiple levels of the biological organization responding to selection is critical (Edelsparre et al. 2024; Urban et al. 2024). In addition to characterizing how relevant phenotypic traits change, it is fundamental to analyze the underlying molecular dynamics. In this context, studying changes in gene expression associated with thermal adaptation adds an important dimension to the understanding of the molecular mechanisms underlying the adaptive process (Sørensen et al. 2007; Porcelli et al. 2016; Mallard et al. 2018; Manenti et al. 2018; Lecheta et al. 2020; Logan and Cox 2020; Oomen and Hutchings 2022). However, there is a lack of studies on the evolutionary dynamics of gene expression, for which multiple time points are needed. We will elaborate on this below.

Experimental evolution is an approach that allows following the real-time evolution of populations as they adapt to a new environment, thus providing a direct measure of their fate under well-designed and controlled experiments (Kawecki et al. 2012). When multiple generations are analyzed, experimental evolution allows characterizing the patterns of the evolutionary trajectories (Simões et al. 2008, 2019; Bailey and Bataillon 2016; Olazcuaga et al. 2021), which is crucial because it allows estimating the rate of adaptation and thus enables to understand how quickly—and through which mechanisms—a population can respond and cope (or not) with new environmental challenges (Kawecki et al. 2012, Santos et al. 2024).

Combining the power of experimental evolution with genome-wide (DNA or RNA) analysis—Evolve and Resequence, E&R (Turner et al. 2011) allows to deepen our understanding of the molecular mechanisms involved in the adaptive dynamics. However, few E&R studies in sexual organisms have characterized several time points during adaptation underlying the temporal dynamics (but see Orozco-Terwengel et al. 2012; Seabra et al. 2018; Barghi et al. 2019; Michalak et al. 2019; Ramirez-Lanzas et al. 2025). In particular, the studies by Orozco-Terwengel et al., (2012) and Seabra et al., (2018), done in *Drosophila melanogaster* and *D. subobscura*, respectively, focusing on genome-wide trajectories during laboratory adaptation (and not thermal adaptation), provide powerful insights into the dynamics of allele frequency changes during adaptation. Both revealed heterogeneous allele frequency trajectories and deviations from classic selective sweep expectations (Messer and Petrov 2013; Hermisson and Pennings 2017), including early plateaus. Seabra et al. (2018) even observed evolutionary reversals in the direction of several single-nucleotide polymorphisms (SNPs). Several theoretical and empirical studies described plateauing patterns (Chevin and Hospital 2008; Burke et al. 2010; Burke and Long 2012; Long et al. 2015; Franssen et al. 2017; Höllinger et al. 2019; Barghi et al. 2020) and underlying mechanisms that could generate them. In another study of laboratory adaptation involving genome-wide evolutionary trajectories in *D. simulans* populations, Barghi et al. (2019) found highly heterogeneous evolutionary trajectories between replicated populations, with adaptation involving multiple genetic pathways despite convergent phenotypic outcomes. Nevertheless, their focus was not on the general temporal dynamics across replicates e.g. that might generate plateauing or reversals in direction. As in Orozco-Terwengel et al. (2012) and Seabra et al. (2018), plateaus were also found in an E&R study analyzing high-protein diet adaptation (Ramirez-Lanzas et al. 2025), in this case addressing changes in the transcriptome and phenotypic traits. This study illustrates the insight obtained by analyzing the temporal dynamics of gene expression through time to understand adaptation to new environments.

Addressing evolutionary changes in gene expression can be a powerful approach to understand adaptive responses to new thermal challenges at the molecular genetic level (Oomen and Hutchings 2022). Linking gene expression changes to those of fitness-related traits has provided important clues about biological processes and specific genes associated with evolutionary responses to temperature (Sørensen et al. 2005, 2007; Laayouni et al. 2007; Mallard et al. 2018, 2020; Manenti et al. 2018; Jakšić et al. 2020; Antunes et al. 2024; Santos et al. 2024). Several candidate genes for changes in gene expression during thermal adaptation have been identified, such as cp, tot genes, and neuronal activity genes in *D. simulans* (Manenti et al. 2018; Jakšić et al. 2020) and Hsps in *D. melanogaster* (Sørensen et al. 2005). It has also been observed that the evolution under higher temperature led to a consistent downregulation of genes from the glycolysis pathway (e.g. Pfk and Eno genes, Mallard et al. 2018). However, as far as we know, there are no studies of the evolution of gene expression during thermal evolution characterizing changes in evolutionary dynamics through time, that is, involving more than a single generation of analysis. In addition, the existing studies involve thermal adaptation to fixed conditions, which may be unrealistic. In changing thermal conditions, selection acts on traits to follow a moving optimum. When this progresses at a constant rate, populations adapt in the same direction but lag behind, depending on factors such as the rate of environmental change, selection intensity, and available genetic variation (Kopp and Matuszewski 2014; Kopp et al. 2018). If populations fail to track this moving target, their persistence may be jeopardized (Bürger and Lynch 1995). The purpose of the study presented here is to fill these gaps (see below).

*Drosophila subobscura* is a valuable model organism to investigate the evolutionary potential of populations in the face of global warming. Both native European and colonizing American populations of the species have clear body size and chromosomal inversion frequency clines, with the latter being consistently linked to temperature variation (Balanyá et al. 2006; Rego et al. 2010; Rezende et al. 2010; Calabria et al. 2012; Rodríguez-Trelles et al. 2013)⁠. Taking advantage of those indications, our team has been studying the real-time thermal evolution of *D. subobscura* populations of distinct biogeographic history (derived from two contrasting European latitudes) exposed to an environment that included an increase in both mean temperature and thermal amplitude (lower daily minimum and higher maximum) between generations, simulating expectations of global warming across generations (details in Santos et al. 2021). The populations that evolved in the warming regime did not reveal an improvement in reproductive success after 22 generations of evolution under such progressive warming conditions, but only detected after 39 generations (Santos et al. 2023). However, the analysis of the transcriptomic evolution of these populations tested in the warming environment revealed a pronounced effect of selection as early as generation 23 (Antunes et al. 2024), with thousands of candidate genes detected as significantly changing expression between experimental populations and their controls, especially in those from the high latitude origin.

In the present study, we specifically focus on the temporal dynamics of transcriptome changes of these populations, analyzing the evolutionary changes after 9 generations and then again after 23 generations (hereafter referred to as G9 and G23) of experimental evolution, to better understand changes through time in evolutionary rate and pattern during the early stages of thermal adaptation. Given that the thermal profile continuously changed between generations in the warming regime, we tested all populations in the ancestral (control) environment, in order to analyze the different generations under a comparable temperature. Moreover, the analysis of the temporal dynamics of gene expression in the ancestral environment is, per se, an important component of the consequences of thermal evolution. As a result, in contrast with Antunes et al. (2024), we are not analyzing evolution in the stressful environment but rather in that of the ancestral populations. With such analysis, we may detect candidate genes that evolve as a correlated response to selection, expressed in the ancestral environment.

**Fig. 1.**
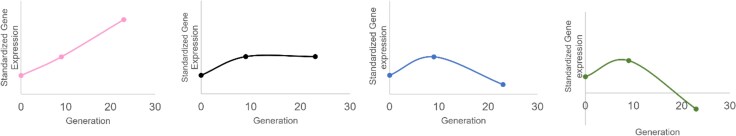
Four possible patterns of change in expression across generations for candidate genes of thermal adaptation: the pink pattern represents genes that show a progressive, directional change across generations 9 and 23 (e.g. upregulated genes steadily increase their expression across generations); the black pattern represents genes that reach a plateau in more advanced generations; the blue and green patterns represent genes that switch direction of change over time (where green represents genes that are upregulated by generation 9 and downregulated by generation 23 or vice versa). Importantly, the same rationale for these patterns can be applied to genes that show initial downregulation.

As noted above, by generation 22, our populations did not show a clear response in life-history traits to the new warming environment. This may indicate that, in this generation, the evolving populations are still in an early phase of the evolutionary response. As such, and considering that populations are responding to a changing environment, a plateauing phase in gene expression is not expected, at least not in such an early phase of evolution. Following this reasoning, we expect that genes that show signs of selection as upregulated at G9 (relative to paired control populations) will continue to increase their expression at G23 (Fig. 1, pink pattern) and the same rationale in the direction of downregulation (i.e. continuing to decrease expression across generations). This will also lead to a higher number of genes detected as candidates at G23 due to the increase in statistical power. These are the patterns expected according to the predictions of classical directional selection (progressive evolutionary pattern, Messer and Petrov 2013; Hermisson and Pennings 2017), even in our case, where the populations are evolving under progressively warmer conditions. More complex, alternative evolutionary patterns (such as those in Fig. 1, blue and green patterns) are likely to be less common. Nevertheless, it is worth testing how consistent the signs and direction of change are across generations, e.g. can a plateau (Fig. 1, black pattern) or switch in the direction of gene expression (Fig. 1, blue and green patterns) occur, e.g. in parallel to Seabra et al. (2018) findings of reverse direction in several candidate SNPs possibly related to epistatic interactions or linkage association changes? Finally, can we associate different evolutionary dynamics with specific functions/pathways, and are they different between populations with distinct histories? By analyzing different populations at different time points of evolution, we aim to better understand the dynamics of gene expression during adaptation to a changing thermal environment. We also seek to determine whether these dynamics differ between populations with distinct evolutionary histories or instead follow a consistent pattern, allowing for broader generalizations across populations.

## Results

### Overall Differential Expression Analysis

We first performed an overall differential gene expression analysis (under the ancestral conditions), of all data from both generations 9 and 23 of our control populations (from Portugal and Netherlands, three-fold replicated—respectively, PT1-3 and NL1-3) and the experimental populations that evolved under a progressively warmer temperature (WPT1-3 and WNL1-3 for the replicated populations from Portuguese and Dutch origin, respectively). We found a huge variation in gene expression between generations—5,325 significant genes for the factor “Generation”; see model 1 in Materials and Methods—while 587 genes showed differences between historical populations (factor “History”) and 90 between thermal selection regimes (factor “Selection”). Importantly, we found 163 genes with significant evolutionary dynamics across generations 9 and 23 (i.e. significant for the interaction Generation × Selection; see Table 1 and Table S1 for more details). The higher number of genes significant for the “Generation” factor is most likely due to environmental differences due to stochastic effects, not being per se relevant for the objectives of the study, where comparisons between experimental and control populations assayed at each generation in synchrony are essential. Based on these results, we decided to separate the analysis of generation 9 and generation 23.

**Table 1:** Number of differentially expressed genes (out of the total 12,827 genes analyzed) and proportion, per factor under test

Factors	1%FDR (*P*-value < 0.001)
Selection	90
Generation	5,325
History	587
Generation:history	389
Generation:selection	163
History:selection	328
Generation:history:selection	134

The analysis of the two generations separately (see model 2 in Materials and Methods) showed that the interaction between Selection and History was significant in a substantially higher number of genes in generation 23 compared to generation 9–1,321 genes versus 294 genes, respectively (Table S2). Based on that high number of genes, we decided to analyze the two historical populations separately, both in each generation (see model 3 in Materials and Methods and Results below), and analysis across generations (model 4 in Materials and Methods).

### Candidate Genes at Generations 9 and 23 and Evolutionary Dynamics Between Generations

After an overall characterization of gene expression variation across generations, we then focused on the detection of candidate genes for thermal adaptation at a shorter (generation G9) and a longer (generation G23) evolutionary time point in each set of replicate populations, comparing at each generation WNL1-3 with NL1-3 and WPT1-3 with PT1-3. In a short period, we identified 343 and 1,145 genes under selection in the set of replicated populations derived from Portugal (WPT1-3) and the Netherlands (WNL1-3), respectively. For the longer period, we identified 1,173 candidate genes in WPT and 1,939 candidate genes in WNL (see Table S3). The overlap (common) candidate genes between generations 9 and 23 were generally low, with only 43 and 199 genes in WPT and WNL, respectively (Fig. S1).

We also targeted genes showing significant differences in expression between generations (see model 4, Materials and Methods). Such differences in the temporal dynamics of the thermal selection regimes (significant term generation × selection) were observed in 127 genes for WPT and 409 genes for WNL (Table S4), with only 6 genes shared between populations of different origin.

### Temporal Patterns of Changes in the Differential Expression of Candidate Genes

**Fig. 2.**
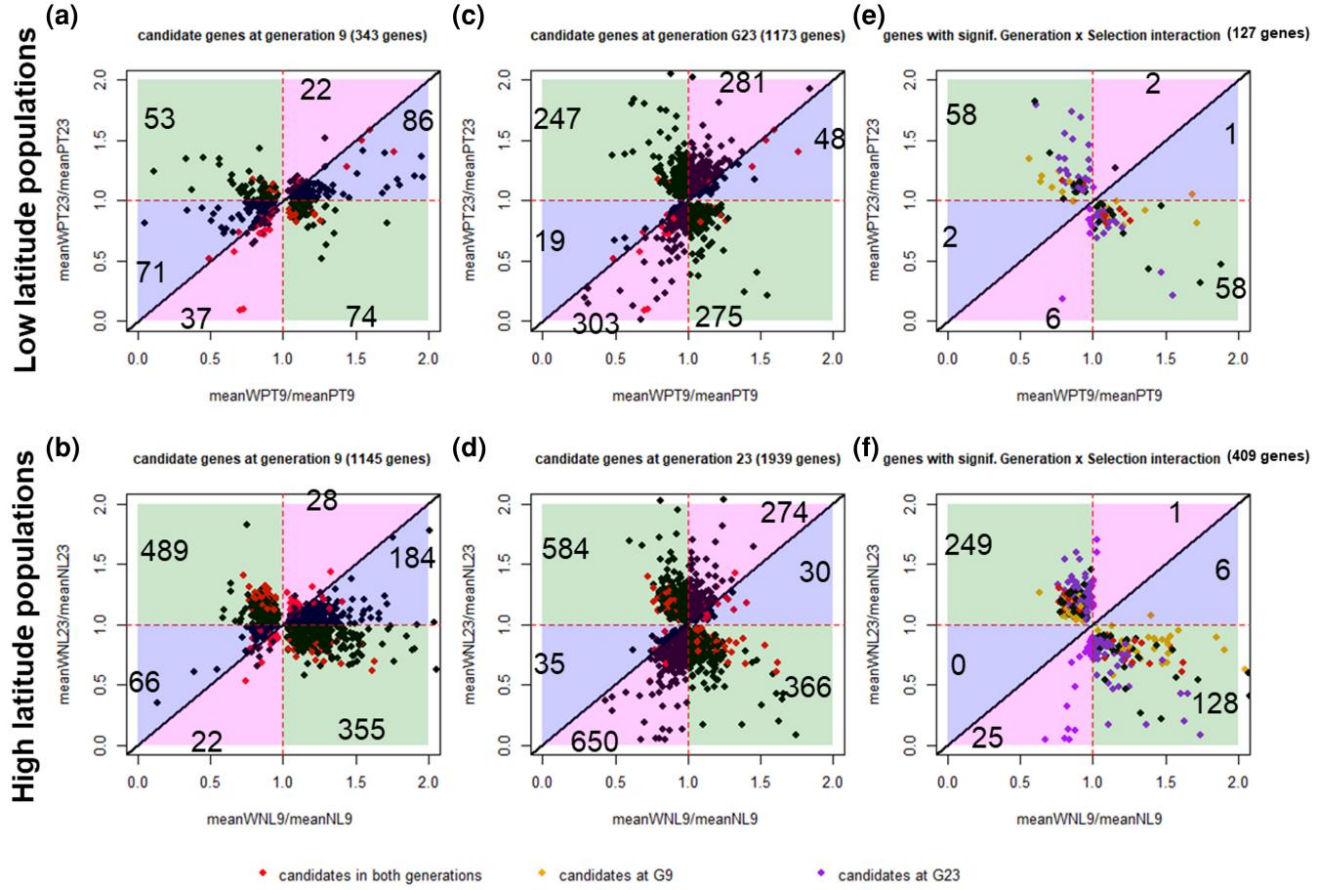
Scatter plots for the dynamics of gene expression evolution. Candidate genes from the Portuguese population (WPT) are shown in the top panels, while those from The Netherlands (WNL) appear in the bottom panels. Panels A and B refer to genes under selection in G9, panels C and D refer to genes under selection in G23, and panels E and F refer to genes showing dynamics (interaction generation × selection). The *x*-axis refers to the ratio of expression between warming and control populations in the 9th generation (e.g. WPT9/PT9) and the *y*-axis represents the same ratio in the 23rd generation (e.g. WPT23/PT23). The numbers in the border of each colored zone correspond to the number of genes in that zone. Red dots are candidate genes that are shared between generations. In the plots E and F, the orange dots are candidate genes at generation 9, and the purple dots are candidate genes at generation 23. See also Fig. 5 for interpretation related to the different coloring of regions.

To gain a deeper insight into the evolutionary patterns across generations of candidate genes detected at each generation (from model 3 in Materials and Methods) as well as those indicating significant changes across generations (from model 4 in Materials and Methods), we examined their temporal changes in the magnitude and direction of differential expression across generations (e.g. from upregulation to downregulation between warming and control populations or vice versa). To compare the response of genes in the first and second time periods, we plotted the ratio of expression between warming and control populations at generation 9 against the corresponding ratio of expression at generation 23 for each set of genes (see Fig. 2, the color coding in this figure corresponds to the patterns shown in Fig. 1. For additional clarification, see Fig. 5 in the Materials and Methods section). It is important to note that this approach represents a simplification. By drawing continuous lines between expression values at discrete time points in Fig. 1, we implicitly assume a smooth trajectory of gene expression change across evolutionary time. However, this may not reflect the true dynamics of the system. In reality, there could be fluctuations in gene expression between the sampled time points (e.g. between generations 0–9 and 9–23).

We first examined how the candidate genes from generation 9 changed by generation 23 (Fig. 2a and b). In WPT, only a small number of candidate genes showed the expected consistent increase (22) or decrease (37) in expression levels between generations, as shown by the pink areas in Fig. 2a. On the other hand, a larger number of genes reduced their difference between generations, slowing down the increase (86) or decrease (71) in expression by generation 23 relative to generation 9 while maintaining the overall sign (see blue areas Fig. 2a). Interestingly, 127 genes even changed their expression signal between generations, from up- to downregulation in 74 genes and from down- to upregulation in 53 genes (see green areas Fig. 2a). For WNL, only 28 and 22 candidate genes showed consistent increase or decrease across generations, respectively (pink area of Fig. 2b), with 184 and 66 genes decreasing the magnitude while maintaining the direction of change in expression across generations (see blue area Fig. 2b). Interestingly, a very large number of genes in these populations reversed the signal of expression between generations (355 with upregulation in warming populations at G9 followed by downregulation at G23, and 489 genes with the opposite pattern, see green area Fig. 2b). WNL also presented a low number of candidate genes in common across generations (199 genes).

We observed a similar pattern when comparing WPT and WNL (compare Fig. 2a with b), with both showing few genes with consistent increases or decreases (pink areas) and most genes with slowing down of changes in expression or even inverted expression across generations (blue and green areas, respectively). However, there are clear differences that are worth noting. In fact, candidate genes in the WNL show a much stronger tendency for a reversal in the direction of gene expression between the warming and control regime across generations—74% of the candidate genes show reversal in WNL (genes in the two green areas of Fig. 2b) and only 37% in WPT (genes in the two green areas of Fig. 2a).

When we analyzed the candidate genes detected by G23 we found that most were not candidates in G9 (black dots vs. red dots in Fig. 2c and d). These genes followed two major patterns: the expected pattern of consistent increase—or decrease (see pink areas Fig. 2c and d) but also the pattern of change in signal (green area of Fig. 2c and d). The number of genes following the green versus pink patterns is much more balanced between populations of different origins than previously observed for the candidate genes detected in G9. In WPT 281 and 303 genes show the pattern of consistent increase or decrease across generations, respectively, and 275 and 247 genes reversed their expression (dots in green areas) while only 48 and 19 maintain the direction, slowing down the change in expression from generations 9 to 23 (dots in blue areas). In WNL 274 and 650 genes show the pattern of consistent increase and decrease, respectively, while 366 and 584 changed their signal across generations (dots in green areas), with only 30 and 35 slowing down their change in expression between generations (dots in blue areas). These results indicate some similarities in the evolutionary dynamics of both WPT and WNL populations, although a general tendency toward downregulation of warming populations by generation 9 can be observed in WNL (the number of genes in the second and third quadrants is clearly higher than in the 1st and 4th—see Fig. 2d), which is not the case for WPT (Fig. 2c).

Analysis of the plateauing patterns—based on candidate genes detected in both generations 9 and 23—identified few genes in both populations, with a total of 14 genes in WPT and 19 genes in the WNL (see Table S5 and Fig. S2).

Finally, we analyzed the patterns of genes that showed significantly different evolutionary dynamics between generations (Generation × Selection interaction)—see Fig. 2e and f. As expected, these were the ones with a higher magnitude of change between generations, including change of direction, from up to downregulation or vice versa. In fact, almost all genes fall into the green areas of Fig. 2e and f, indicating a reversal of expression between generations. In these analyses, we found consistent dynamics between populations with different historical backgrounds as the dots/genes fall in the same-colored areas, even if the absolute number of genes is much higher in WNL. However, as for candidate genes at G23, the number of genes in the second and third quadrants is clearly higher than in the 1st and 4th quadrants, which is not observed in WPT(Fig. 2e vs. Fig. 2f).

In brief, in the first column of Fig. 2 (panels A and B), there is a small number of dots (genes) with consistent changes in direction between generations, i.e. steady increase or decrease (areas colored in pink). In the second column (panels C and D), there is a clearly small number of genes that have decreased their absolute expression (areas colored in Blue), and in the third column (panels E and F), there is an extremely high number of genes that reverted patterns, from up to downregulation or vice versa (areas colored in Green).

### Biological Insights of Candidate Genes in Both Generations

We next pursued a functional characterization of the candidate genes for thermal adaptation with the most relevant evolutionary dynamics. Candidate genes in both generations—red dots in Fig. 2—that showed consistency (pink pattern of change) or reversal of expression change across generations (green pattern of change)—see Table S6—were characterized in terms of biological processes. These genes were chosen due to their highly contrasting expression patterns, providing valuable insights into the underlying mechanisms driving gene expression dynamics during adaptation.

Candidate genes that show a reversal of expression change across generations are enriched for processes associated with DNA replication in both populations (see Fig. S3). Genes with a reversal pattern were significantly enriched for DNA recombination and repair in WPT and for the toll-NF-kappaB and EGFR signaling pathways only in WNL (Fig. S3 and Table S7 for more details). Genes with consistent directional changes across generations (Pink patterns) were not significantly enriched for any functional process, likely due to the low number of candidate genes in both generations that follow this pattern (see Table S7).

We decided to investigate further the genes involved in the toll-NF-kappaB sgnaling pathway. This enrichment was caused by two genes—wek and Dif—that presented opposite patterns of up and downregulation between generations (see Table 2) which explains why the toll-NF-kappaB signaling Pathway is not enriched when the patterns are analyzed independently—see below. Interestingly, when analyzing the patterns of the core component genes of the toll-NF-kappaB signaling pathway, we found that eight of ten genes were downregulated in generation 23 of WNL, with four of these showing significant effects of selection when tested in environment C (see asterisk in Table 2). Strong evidence of evolutionary downregulation was also observed in the environment W (Antunes et al. 2024). Curiously, downregulation of Toll genes was less pronounced in generation 9, with three genes showing such a pattern in WNL (see Table 2).

**Table 2:** Up or downregulation of toll-NF-kappaB signaling pathway core genes in the warming populations by generation 9 and 23

Gene symbol D. mel (gene ID D. sub)	Portuguese population			Dutch population		
	G9 Environment C	G23 Environment C	G23 Environment W	G9 Environment C	G23 Environment C	G23 Environment W
Dif (LOC117902321)	Down	**Down** ^a^	Up	**Up** ^a^	**Down** ^a^	Down
wek (LOC117898393)	Down	**Down** ^a^	Down	**Down** ^a^	**Up** ^a^	Up
Tl (LOC117896380)	Up	Up	Up	Down	**Down** ^a^	**Down** ^a^
NT1 (LOC117893050)	Down	Up	Up	Up	Down	Up
Dl (LOC117900308)	Up	Down	Up	Down	**Down** ^a^	**Down** ^a^
Toll-7 (LOC117890578)	Down	Up	Up	**Down** ^a^	Down	**Down** ^a^
Myd88 (LOC117890658)	Up	Up	Up	Up	**Down** ^a^	**Down** ^a^
pll (LOC117897415)	Down	Up	Down	Down	Up	Down
tub (LOC117897755)	Up	**Down** ^a^	Down	**Down** ^a^	Down	Down
Spz (LOC117899350)	Up	Up	Up	Down	Down	**Down** ^a^

The first two columns of each population of different origin show the direction of expression tested in the control environment, while the third column shows the direction of expression tested in a warming environment (see Antunes et al. 2024).

^a^Indicates statistical significance for the selection factor.

**Fig. 3.**
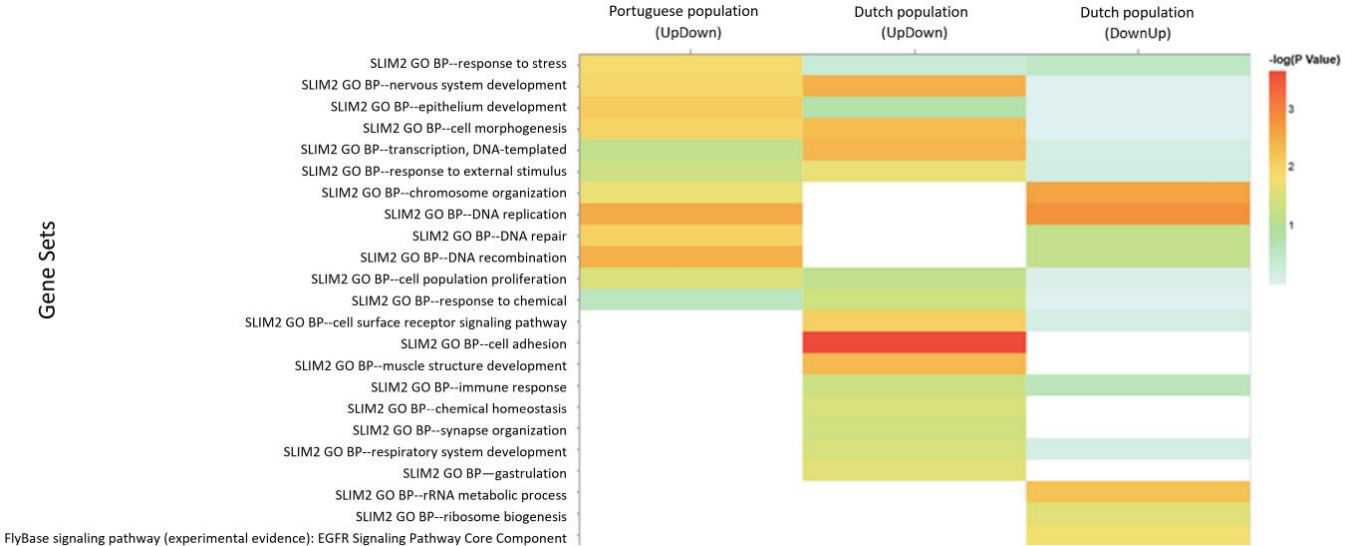
Heat map of the functional characterization of genes with reversal patterns across generations (red dots in green areas of fig. 2) separated by quadrant. The first column refers to genes from the Portuguese population (WPT) that fall into the fourth quadrant showing upregulation followed by downregulation (see Figs. 2 and 5 for further enlightenment). The second column and third columns refer to genes from the Dutch population (WNL) that fall into the fourth quadrant, showing upregulation followed by downregulation (third column), and that fall into the second quadrant, showing downregulation followed by upregulation. The color gradient indicates –log(*P*-value).

Despite the biological insights gained from the functional characterization of genes with reversal patterns, interpretation is limited by the fact that reversal patterns may reflect two different dynamics (associated with two quadrants in Fig. 2): one of upregulation by G9 followed by downregulation by G23, and one of downregulation followed by upregulation. To distinguish between these two dynamics, enrichment analysis was applied to these two sets of genes separately. We found that processes of DNA replication, recombination, repair, and epithelium development are first upregulated and then downregulated in WPT. These processes share several genes in common with other functions, such as “stress response.” For example, genes such as Caf1-180, Ago, and Nipped-B are included in several of these categories. In WNL, processes such as cell adhesion, morphogenesis, nervous system development, and others are first upregulated and then downregulated. Interestingly, in WNL, we found that DNA replication and repair functions are first downregulated and then upregulated in more advanced generations, suggesting that the timing of the upregulation of these mechanisms involved in DNA replication and repair does not align with WPTs. In addition, the Pathway EGFR was found to be first downregulated and then upregulated in WNL (Fig. 3 and Table S8). This pathway is usually activated in stressful situations associated with regeneration processes.

## Discussion

In this study, we examine the evolutionary dynamics of gene expression in replicated populations derived from two historically differentiated populations of *D. subobscura* (Portuguese and Dutch) evolving under an increasingly warming environment. Analyzing gene expression at generations 9 and 23, we focus on the timeframe where we applied an increase of both mean temperature and daily amplitude every generation. We provide evidence for an incremental impact of selection at the genome-wide gene expression level across generations, with a higher number of candidate genes differing between selective regimes after 23 generations than after only 9 generations of evolution. This indicates, as expected, that prolonged exposure to increasing warming conditions is associated with more extensive evolutionary changes at the transcriptomic level, including broad changes in gene expression. Importantly, the low overlap of candidate genes between generations suggests a dynamic and changing genetic landscape, contrary to what we expected at such an early phase of adaptation. This finding indicates that different sets of genes are recruited over time, even within such a short period of time, in response to the increasing environmental stress. This is also linked to the limited presence of clear plateauing patterns in gene expression—a feature that may be explained by the relatively short duration of the experiment as well as the fact that populations are evolving in an environment with changes in temperature every generation. Differences were found between populations of contrasting initial history, with greater evidence of more complex patterns in the Dutch population, as well as contrasting processes underlying gene expression changes (see below).

An important note is in order: in this study, we analyzed the evolution of gene expression in the ancestral environment, and not in the new, warming environment where selection is acting directly. One practical reason was the fact that the warming regime is a dynamic one, with a planned rise in mean temperature and amplitude throughout generations, conditional on their capacity to endure such changes. This made it difficult to predict what would be the stressful temperature to test at different generations for a temporal dynamics analysis. This is because it is essential to use the same environment across generations for the interpretation of evolutionary dynamics as a function of underlying genetic changes across generations. It is thus convenient to use the ancestral environment as the target of a temporal analysis, since it remained stable across generations, while the warming environment did not. Moreover, the evolutionary dynamics expressed in the ancestral, non-stressful environment is by itself of most relevance, since it allows to characterize the evolutionary divergence due to adaptation to novel, stressful challenges. Having said this, it is important to consider that in this study we are detecting correlated evolutionary changes that cannot be directly extrapolated to those expressed in the warming environment, though they are a consequence of that evolution.

### Pervasive Complex Dynamics of Gene Expression During Thermal Evolution

Our aim here was to investigate the dynamics of evolutionary changes as expressed in the ancestral environment, by comparing transcriptomic changes during the first 9 generations and those after 23 generations. The comparative analysis of these two periods is particularly interesting given that by ∼ generation 23 (under warming conditions), we found pervasive selective response at the transcriptome level, though not at higher order traits (reproductive success) (Santos et al 2023, Antunes et al. 2024). This suggested that gene expression changes preceded those at higher-order traits, though they may also be due to higher statistical power/sensitivity to infer adaptive dynamics at the gene expression level.

Under a directional, progressive evolutionary pattern, we would expect a higher number of genes to be detected by generation 23, given that increased changes in gene expression would allow for a stronger sign of selection. Here we found that, in the Portuguese population, 584 genes showed the progressive pattern at generation 23, compared to only 59 genes at generation 9, and an even greater difference was found in the Dutch population (924 at generation 23 vs. 50 at generation 9). However, when we analyzed in more detail the evolutionary patterns underlying adaptation to warming temperatures, we found a more complex scenario with substantial non-directional changes in gene expression even in this early phase of adaptation, which has not yet driven evident evolutionary changes in higher order, life-history traits (see Santos et al. 2023). Given the progressively warmer temperatures, the simplest scenario would be of a steady directional change in gene expression. Nevertheless, this experiment included daily fluctuating temperature conditions with increments in both low and high peak temperatures across generations (e.g. Santos et al. 2021). Considering this, it is possible that gene expression responses are more dynamic and complex, namely with some genes showing shifts from up to down regulation across generations and vice versa. In fact, some studies have also demonstrated that populations adapted to high and low temperatures often rely on distinct loci (Michalak et al. 2019; Otte et al. 2021). This indicates that the biological mechanisms involved in adapting to heat versus cold—or to gradual temperature increases—may differ significantly, which may impact the transcriptome dynamics, namely involving regulatory pathways (López-Maury et al. 2008; Harrison et al. 2012). These complex dynamics may also be influenced by changes in population size, likely to occur during evolution under harsh environments. Notably, the warming populations suffered a steep decline in census size by generation 22, followed by a quick recovery by the assayed generation 23 (Table S9). These census size changes might have affected the evolutionary dynamics of our populations, e.g. by reducing the evolutionary response after the bottleneck. However, given that such census changes occurred at the very end of the analyzed time frame and were not prolonged, we do not expect high deviations from a monotonic pattern. Of the candidate genes identified at generation 9, few also showed significant evidence of selection at generation 23 (43 and 199 genes in the Portuguese and Dutch population, respectively), and accordingly, few showed consistent changes in expression across generations. Specifically, in the Portuguese population, only 6% of genes maintained consistent up- or downregulation, while a significant number (83% in total) showed changes contrary to initial expectations, slowing or reversing the direction of their expression across generations. The Dutch population showed even greater variability, with 96% of genes showing changes contrary to expectations, slowing or reversing their expression change (see Fig. 2a and b). In contrast, by generation 23, there was an increase in the number of genes that consistently increased their expression (pink areas, Fig. 2c and d). This latter class of genes was not recruited as candidate genes by generation 9 due to their (still) low expression levels in this early generation. However, there is a high number of genes with non-directional dynamics, which is more pronounced in the Dutch population, with a predominant pattern of initial downregulation followed by upregulation. The prevalence of such complex dynamics suggests a potential instability in adaptive gene expression or a strategic temporal flexibility that allows populations to cope with changing thermal conditions (Danks 1991). The fact that this pattern is more prevalent in the Dutch population suggests a more complex adaptive process in this population. The Dutch population experiences more pronounced evolutionary change, presumably because they are further from an optimal adaptive state (e.g Ghalambor et al. 2007, 2015; Morris and Rogers 2013; Gibert et al. 2019). A similar trend of greater evolutionary changes in gene expression in the Dutch population was observed when they were specifically tested in the warming environment by generation 23 (Antunes et al. 2024).

### Comparing Genomic and Transcriptomic Evolutionary Dynamics

There is a lack of studies on the evolutionary dynamics of the transcriptome in populations evolving under novel thermal challenges, which would be important to scrutinize temporal molecular changes. Here, we propose to address this gap by comparing gene expression levels in different generations of thermal evolution. We found strong reversal patterns in gene expression between generations, a phenomenon that has not been previously documented as associated with adaptation to warming. However, it is important to note that our analysis is based on only two temporal periods—the first 9 generations and between generations 9 and 23. This calls for caution in generalizations to the full trajectory of the dynamics of gene expression across those generations. In any case, our main aim is to analyze whether the sign of the evolutionary response changed direction or not between periods, which does not require additional data. It is tempting to draw comparisons with other studies targeting the dynamics of adaptation (e.g. (Orozco-Terwengel et al. 2012; Seabra et al. 2018; Barghi et al. 2019; Langmüller and Schlötterer 2020; Phillips et al. 2020), even though these focus on genomic variation. Below, we address this, although such comparisons need to be taken with caution since studies have shown that linking genome and transcriptome evolutionary patterns is a complex task (see Dettman et al. 2012 for a review).

According to Seabra et al. (2018), the reversed patterns of changes in the frequencies of the candidate SNPs they analyzed may be caused by epistasis or changes in linkage association with a decrease in selection coefficient (Chevin and Hospital 2008, Franssen et al. 2017). This could also be the case for the reverse patterns observed for gene expression changes shown in this study, with the added factor that the changing environmental conditions that we imposed (which were not present in the Seabra et al. 2018 study) could potentially favor large changes in the selection coefficients. In an experimental evolution study over 60 generations in *D. simulans* populations, Langmüller and Schlötterer (2020) also showed that selection signatures identified early in experimental evolution may not reflect the long-term adaptive architecture. Our results are consistent with this, as candidate genes identified at generation 9 did not show consistent trends at generation 23. This may be due to the relatively short duration of the study and/or the possibility that cis and transregulation on gene expression (Osada et al. 2017) confer a higher probability of complex changes in the transcriptome as compared to the genome. In general, it is important to acknowledge that genetic drift may have introduced some temporal variation in gene expression changes and partially contributed to the detected selective responses, as stochastic processes can occasionally produce consistent patterns across replicates. Nonetheless, it is not likely that highly consistent changes of expression due to drift occur across replicates. Future research integrating genomic and transcriptomic analyses will be essential to unravel the complexity of the adaptive process.

### Can we Relate Complex Patterns With Specific Functions/Pathways?

Complex expression patterns, with changes in the direction of gene expression, were widespread at an early stage of the evolutionary response to warming temperatures, which was not expected. Genes showing such shifts were significantly enriched for DNA replication processes in both Portuguese and Dutch populations, with most suggesting an important role for DNA repair in adaptation to warming temperatures—one gene in the Portuguese population (Caf1-180) and three genes in the Dutch population (spn-A; RPA2 and RfC4). Notably, the timing of these upregulation events differed: the Portuguese population upregulated DNA replication mechanisms and DNA repair earlier (in generation 9), followed by downregulation later, whereas the Dutch population showed the opposite pattern. This suggests that Portuguese populations prioritize an initial stress response to avert cellular damage, which is gradually reduced as slower adaptive mechanisms come into play.

In contrast, the Dutch population showed unique enrichment for Toll-NF-kappaB signaling pathway (genes wek and dif) and the EGFR signaling pathway. The Toll-NF-kappaB pathway likely reflects the activity of the immune system (discussed below), while the enrichment for the EGFR signaling pathway, which is associated with stress and tissue regeneration, is potentially associated with an increased need for repair mechanisms in harsher environments (Chou et al. 2014; Hsieh and Pearlman 2015). We observed a widespread trend of downregulation in the core genes in the toll-NF-κB pathway, particularly in the Dutch population. This finding suggests that elevated temperatures can alter the immune systems of insects and underscores the link between immunity and plastic and evolutionary responses to thermal stress (St. Leger 2021). It is not yet known how adaptation to heat affects the immune system. A notable exception is the study of thermal adaptation in *D. melanogaster* by Hsu et al. (2020), which reports the downregulation of immune-related genes, consistent with our findings (see also Junprung et al. 2024) for a similar finding in brine shrimps). One possible explanation for the observed downregulation is the existence of physiological trade-offs, whereby resources are diverted from immune function (Ferguson and Adamo 2023). It is also possible that prolonged heat stress has led to immune depression. However, the fact that the Toll pathway in this species also plays a critical role in embryonic development (Valanne et al. 2011) may complicate our interpretation as our study focused on non-virgin female flies and the abdomen was not removed. This possibility should be further investigated in the future.

### Concluding Remarks

Our study shows an incremental effect of thermal selection on gene expression across generations, with the number of candidate genes responding to selection substantially increasing between generations 9 and 23. This suggests that continued exposure to rising temperatures leads to broader gene expression changes. Moreover, the low overlap in candidate genes across generations suggests a highly dynamic and shifting genetic architecture. A more detailed analysis reveals that these transcriptomic responses are marked by complex and heterogeneous patterns. In fact, gene expression during thermal adaptation involves diverse evolutionary dynamics, even in short-term evolution under a changing environment, with patterns including consistent up- or downregulation, slowing of changes, and even reversals in gene expression. These dynamics differ significantly between the populations of Dutch and Portuguese origin, with the former showing more complex adaptive responses. This is consistent with previous studies suggesting that longer evolutionary times are required for plateauing patterns to become prominent, to which the temperature increase across generations likely contributed. Taken together, these findings suggest a shifting evolutionary landscape and ongoing adaptation to warming conditions, even when conspicuous changes in higher-order traits are not (yet) observable.

## Materials and Methods

### Population Maintenance and Thermal Selection Regimes

In 2013, 213 and 170 female flies were collected in Adraga (PT, Portugal) and Groningen (NL, The Netherlands), respectively, and brought to the laboratory. Shortly thereafter, by generation 4, the populations were threefold replicated (PT1-3 and NL1-3). We often refer to the two historical populations as “Portuguese population” or “Dutch population” to improve readability, although all the analyses were done in the three replicates together. The replicated populations were maintained in discrete generations with a synchronized 28-day cycle, a 12L:12D photoperiod, and a constant temperature of 18°C and egg collections of 70 eggs at the time of the peak fecundity each generation. Throughout 6 years, census sizes were generally maintained between 500 and 1,200 individuals with no relevant bottleneck occurrences (Simões et al. 2017). The stronger phase of lab adaptation produced only very mild declines in the genetic diversity of our populations: between generations 5 and 26 pi declined from 0.096 to 0.093 in NL populations and from 0.092 to 0.084 in PT.

**Fig. 4.**
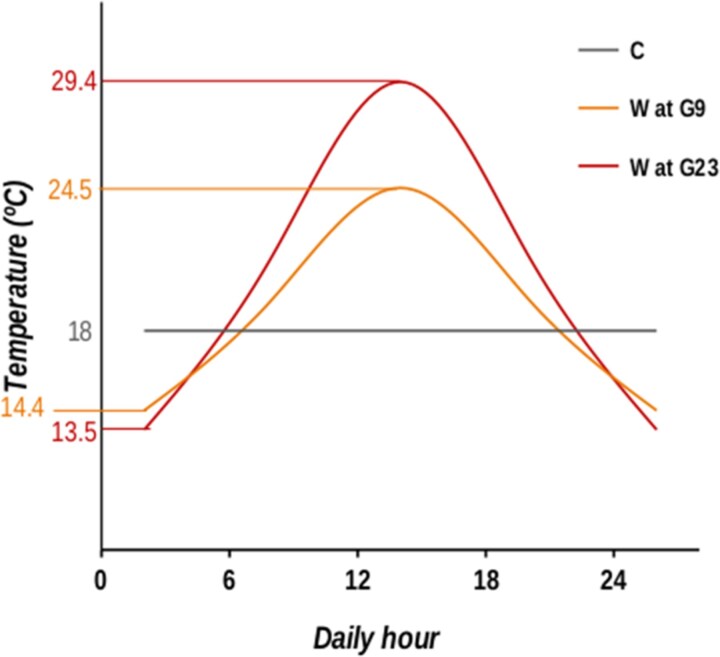
Daily temperature in the two thermal regimes: control regime (constant 18°C) and global warming regime (W). In the W regime, the first-generation experienced temperatures between 15 and 21 °C, the 9th generation experienced temperatures between 14.4 and 24.5°C and the 23rd generation experienced temperatures between 13.5 and 29.4°C.

In 2019, the thermal experimental evolution started, with the implementation of a new, warming selection regime in both PT and NL populations, generating the WPT and WNL populations, respectively. This regime introduced daily temperature fluctuations, starting between 15 °C and 21 °C in the first generation, with a gradual increase in both daily mean (0.18 °C) and amplitude every generation (0.54 °C), exhibiting minimal decreases and maximal increases (Fig. 4 and Santos et al. 2021 for details). At the same time, the ancestral PT and NL populations served as controls (C), maintained at their constant temperature of 18 °C (see more details below).

Upon reaching the 22nd generation and a maximal daily temperature of 30.2 °C, the increases in the thermal mean and amplitude of the global warming regime had to be halted due to low viability, resulting in a significant decrease in the census size of the populations and generating a bottleneck (see Table S9). Subsequently, the populations in the warming regime were maintained within a temperature cycle that ranged from 13.5 ^o^C to 29.4 ^o^C with a mean temperature of 21.4 ^o^C, which is that of generation 20 (Fig. 4)—see (Santos et al. 2023). Importantly, between generations 22 and 23, the census size increased around 2 to 4 fold, allowing for some recovery.

### Experimental Design

The populations were studied at two time points: after 9 and 23 generations (i.e. how they changed between generations 0 and 9, and between generations 0 and 23) of evolution in the warming selection regime. Since our analyses are always based on comparisons between the warming populations and their controls (representing their ancestors), transcriptomic data were not obtained for generation 0. As we did not know, by generation 9, what temperatures would eventually be reached over the course of the experiment, we analyzed RNA samples only at the control temperature. Prior to each generation analyzed, a common garden environment was used in which all populations completed one full life cycle at 18 ^o^C, i.e. the control temperature. After the common garden phase, the eggs laid by these females were transferred to the control environment, where they developed into adults and gave rise to the samples analyzed (details below). Because it is not possible to analyze samples from different generations simultaneously, we instead compare each experimental population to its respective control population at the same generation, which serves as a proxy for its ancestral state, given the common origin of control and warming populations. The control populations have been in the lab for many generations, and are likely in genetic equilibrium by the time this experiment started, enabling their use as controls to account for the environmental variation between generations. The samples from generation 23 were previously used in Antunes et al. (2024) only for the analysis of thermal plasticity of warming populations, as in that study, the analysis of candidate genes involved only samples developed under warming conditions.

In this study, 24 samples were examined, representing the two selective regimes (control vs. warming), the two histories (of Dutch vs. Portuguese origin), the three replicate populations in each regime and history (e.g. NL1-3), and two generations (generation 9 vs. generation 23). Gene expression changes were always assessed relative to the control population at the corresponding generation. The experimental design involved analyzing pools of 45 adult females from each population-generation combination. These individuals were flash frozen in liquid nitrogen—and subsequently stored at −80 °C—on their 8th day of adult life, a critical point chosen because it corresponds to the age of egg collection for the following generation in the maintenance protocol of both the control and warming regimes. To ensure accuracy, sex screening was performed using CO_2_ anesthesia 2 days before the flash freeze procedure. All sampled females from the same generation were uniformly frozen on the morning of the same day to ensure consistency in the experimental schedule.

### RNA Extraction, Sequencing and Preprocessing of Reads

The TissueRuptor (Qiagen) was used to disrupt the whole bodies of each pool of 45 female flies. The RNeasy Plus Mini kit (Qiagen) was then used to extract total RNA from the pools of females. Nucleic acid quantification was performed using Nanodrop, and RNA integrity was confirmed by agarose gel electrophoresis. Sequencing was performed by Novogene using Illumina SBS technology with rRNA removal protocol and strand-specific libraries, aiming at 100 million reads (or 50 million paired-end reads) of 150 bp (15 Gigabases) per sample. After sequencing, the quality of the paired-end raw reads was assessed using FastQC v0.10.1 (Andrews 2010). Reads were preprocessed using fastp version 0.21.1 (Chen et al. 2018) to trim/remove the poor quality reads, using an average quality value of 20 and a minimum read length of 120 base pairs (bp) which corresponds to 80% of the read length The default value of 5 was used for the n_base_limit parameter which corresponds to the limit of N (not called bases) in the read.

In total, 2,807,301,076 raw reads (pair1 and pair2) were generated, with an average of 116,859,632 reads per G9 sample and 117,082,124 reads per G23 sample, each spanning 150 bp. After elimination of low-quality bases using fastp, 2,787,299,530 reads (99%) were retained for subsequent analyses.

### Mapping of the Reads Against the Reference Genome and Features Assignment

Reads from the 24 samples were mapped to the reference genome of *D. subobscura*—assembly UCBerk_Dsub_1.0—(Bracewell et al. 2019)⁠ using STAR software version 2.7.9a with default parameters (Dobin et al. 2013). The STAR software was executed in a 2-pass mapping configuration. The assignment of the reads to features (genes) was performed using FeatureCounts version 2.0.0 (Liao et al. 2014)⁠ with the parameters “−p” indicating pair-end sequencing, “-F GFF” the annotation format, “-t gene” the feature type to count, “-g ID” the attribute to be identified, “-s 2” the sequencing protocol with dUTP second strand marking, “−C” the non-chimeric only count and “−a” the annotation file. A total of 1,226,371,284 G9 reads and 1,285,842,230 G23 reads mapped successfully to the reference genome, averaging 102,197,607 reads per G9 sample and 107,153,519 reads per G23 sample, with 1,135,133,576 (G9) and 1,193,622,072 (G23) mapping only once (on average 94,594,465 and 99,468,506 reads per G9 and G23 sample, respectively) and denoted as unique mapped reads (UMR)—see Table S10. FeatureCounts allowed us to identify 14,306 genes, representing 99% of the genes in the reference genome.

### Differential Expression Analyses

After selecting the read count files to be used in each analysis, the DESeq2 median of ratios normalization method (Love et al. 2014) within Galaxy (Afgan et al. 2018, Galaxy Version 2.11.40.7 + galaxy2) was used to normalize the read counts. Independent normalization protocols were generated for each analysis, as these involved different samples (see below, models 1-4). The normalized expression counts of each transcript (gene) were used as raw data in the analyses. Only genes that have data in at least 3 of the samples (more than 11,000 genes in each analysis—see Table S11) were used in the analyses. Detection of differential expression was performed for each gene using the glmmTMB R package (Brooks et al. 2017) and models: (1) including all samples; (2) for each generation separately, including data from both historical populations; (3) for each generation and historical population separately; and (4) for each historical population including data from both generations (see details below).

1. Expression ∼ Generation*Selection*History + (1|AP{History})
2. Expression ∼ Selection*History + (1|AP{History})
3. Expression ∼ Selection + (1|AP)
4. Expression ∼ Generation*Selection + (1|AP)

with Expression being the normalized RNA expression values for each gene; Selection, the fixed factor representing thermal selection regimes (categories Control and Warming selection regimes); Generation, the fixed factor representing generation (categories 9 and 23 generations); and AP the random factor that corresponds to the ancestral population (PT1-3, NL1-3) nested in the fixed factor History (e.g. PT low latitude and NL high latitude). This term reflects the extent of replication in our experimental evolution study, capturing both the variability among replicate populations within each thermal condition and the paired structure arising from their common lineage (e.g. NL1 and its descendant WNL1, both originating from the NL background, with WNL1 nested within NL). Since there are only two time points (“generations”) in our analysis, we did not define “generation’ as a numerical variable, as it gives equivalent results to being defined as a factor for most relevant tests.

Akaike information criterion (AIC) tests on the model 1 with and without random factor interactions—see Table S12—were performed to assess the best model (accounting for interactions with the random factor and distribution to be applied). Specifically, AIC tests were performed for each gene to assess the improved fit of the model using two distributions for count data (negative binomial and Poisson). The model-distribution pair finally selected for all genes (excluding interactions with the random factor and the negative binomial distribution) was the one that performed better (had a lowest AIC) for the majority of the genes tested (Table S13). The best model and distribution were taken as the most appropriate for all subsequent analyses. *P*-values were corrected with False Discovery Rate (FDR for alpha = 0.01, *P*-value of 0.001) calculated as follows: $0.01/\left(\sum_{i=1}^{n}\;(1/i)\right)$ where *n* is the gene count (Benjamini and Yekutieli 2001; Theorem 1.3). This rather stringent criterion was applied to reduce the detection of false positive candidate genes for thermal adaptation, by targeting genes with the most consistent patterns across replicates (unlikely to result from drift effects).

### In-Depth Analysis of Gene Expression Patterns Across Generations

**Fig. 5.**
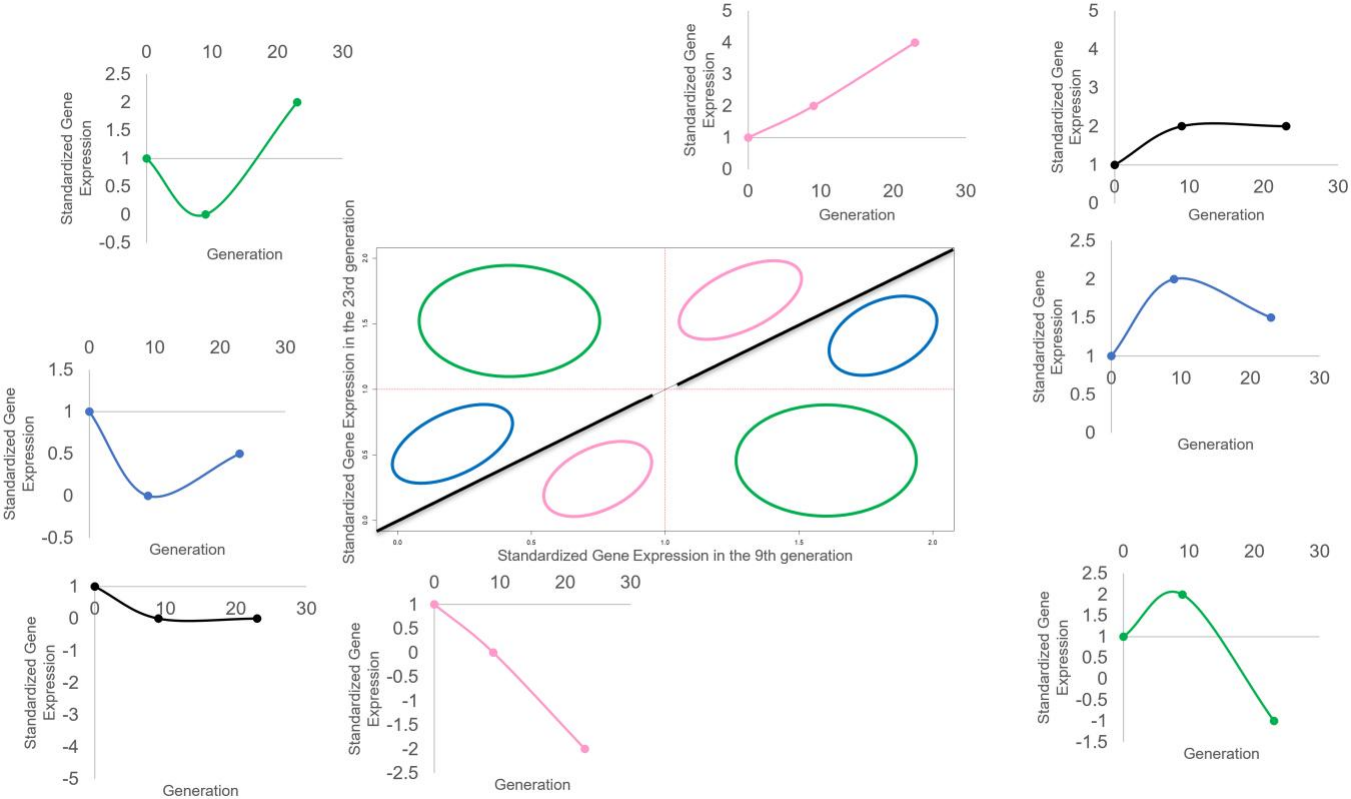
Position occupied by genes with different expression patterns. In the central plot, the *x*-axis refers to the ratio of expression—or standardized expression—between the warming and control populations in the 9th generation (e.g. WPT G9/PT G9) and the *y*-axis represents the same ratio in the 23rd generation (e.g. WPT G23/PT G23). Genes that show a coherent change over time (upregulated, increasing their expression over time or downregulated, decreasing their expression over time) fall over the pink regions; genes that reached a plateau between generations 9 and 23—black pattern—are positioned over the diagonal line of the central plot; genes that decreased their expression (in absolute value) between generations 9 and 23 (while maintaining their sign)—blue pattern—fall into the blue regions of the central plot and genes that were upregulated at generation 9 and became downregulated at generation 23 or vice versa—green pattern—fall into the green regions of the central plot. The different expression patterns (subplots) are arranged around the central plot. Each subplot is color-coded to match the ellipses in the central plot.

Having defined candidate genes by comparing warming and control populations, at each generation, we assessed how the pattern of expression of these genes changed between the two generations. To do this, we plotted the ratio of gene expression between the warming and control populations at generation 9 (e.g. WPT G9/PT G9) against the equivalent ratio at generation 23 (e.g. WPT G23/PT G23) (the R script used to construct the plots is available at https://github.com/marta-antunes/transcriptome_dynamics_G9G23). This allowed us to assess not only whether gene expression changed across generations (information already obtained from model 4), but also how it changed. This was done for four sets of genes: (1) genes detected to be under selection at G9; (2) genes detected to be under selection at G23; (3) genes detected to be under selection at both G9 and G23; and (4) genes with a significant change in the difference between warming and control populations between generations (significant interaction Generation × Selection). Figure 5 illustrates the different patterns expected in the overall plot (in the middle), as well as the different expression dynamics of candidate genes (surrounding the central plot), with different colors highlighting such different patterns (e.g. pink represents continuous directional expression across generations, while black represents gene expression plateaus between generations). We also tried to define the genes that showed a plateauing pattern. We defined those genes as meeting the following conditions: (1) are candidates at both G9 and G23; (2) the interaction Generation × Selection is not significant; (3) whose absolute difference between the ratio in the 23rd generation and the ratio in the 9th generation is less than 0.05 (5% of temporal change).

### Gene Set Enrichment Analysis (GSEA)

We used Proteinortho (Lechner et al. 2011) within Galaxy (with default settings) to detect *D. melanogaster* orthologs for *D. subobscura* proteins, as there is more information on the *D. melanogaster* genome. 8% of the *D. melanogaster* proteins had no equivalent in *D. subobscura*. The “Gene” option in the Batch Entrez tool from NCBI (https://www.ncbi.nlm.nih.gov/sites/batchentrez) was used to obtain the gene symbols for each locus accession. We then performed a Gene Set Enrichment Analysis (GSEA) with PANGEA (PAthway, Network and Gene-set Enrichment Analysis (Hu et al. 2023) using four input gene lists with genes from either Portuguese and Dutch origin that are candidates in both generations and that show either (consistent or reversal of change (pink or green areas, respectively, see gene lists A–D in Supplementary Material Table S6). This web application allows exploring gene lists and highlights common biological features by statistically defining significantly over- or under-represented gene classes relative to predefined gene sets. PANGEA allows the use of other gene sets of shared properties other than Gene Ontology (GO) (e.g. Flybase, KEGG, REACTOME, and others). We chose to perform GSEA based on a subset of Gene Ontology (GO) terms—the Slim2 GO BP—and FlyBase signaling pathway, as our interest extends beyond just Biological Processes to include gene pathways as well. We defined statistically significant features at *P*-values lower than 0.05. We also performed additional GSEA analyses by quadrant for the candidate genes of the green areas to address functions/pathways with the two reversal patterns observed (quadrants 2 and 4, see Figs. 2 and 5).

## Supplementary Material

evag033_Supplementary_Data

## Data Availability

The sequence data for all samples are available at NCBI BioProject PRJNA1161223. Customized scripts used are available in GitHub: https://github.com/marta-antunes/transcriptome_dynamics_G9G23
