# Supplementary material for: Evolutionary Dynamics of Gene Expression During Thermal Adaptation in Drosophila subobscura
Source: Genome Biol Evol. 2026 Mar 24;18(3):evag033. doi: 10.1093/gbe/evag033 (PMC13010818; doi:10.1093/gbe/evag033)
Supplement: evag033_Supplementary_Data [file evag033_supplementary_data.zip › Figures_SuppMaterial_4JAN.docx]

Supplementary Figures


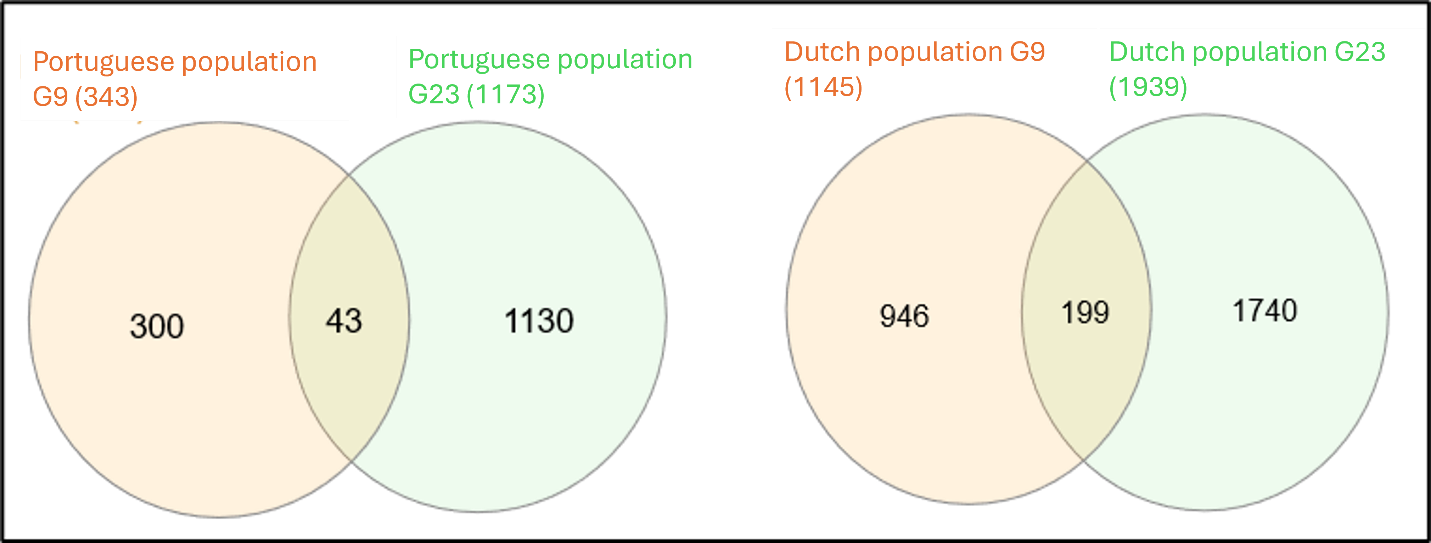


Figure S1 - Overlap of candidate genes between generations 9 and 23 in Portuguese and Dutch populations.


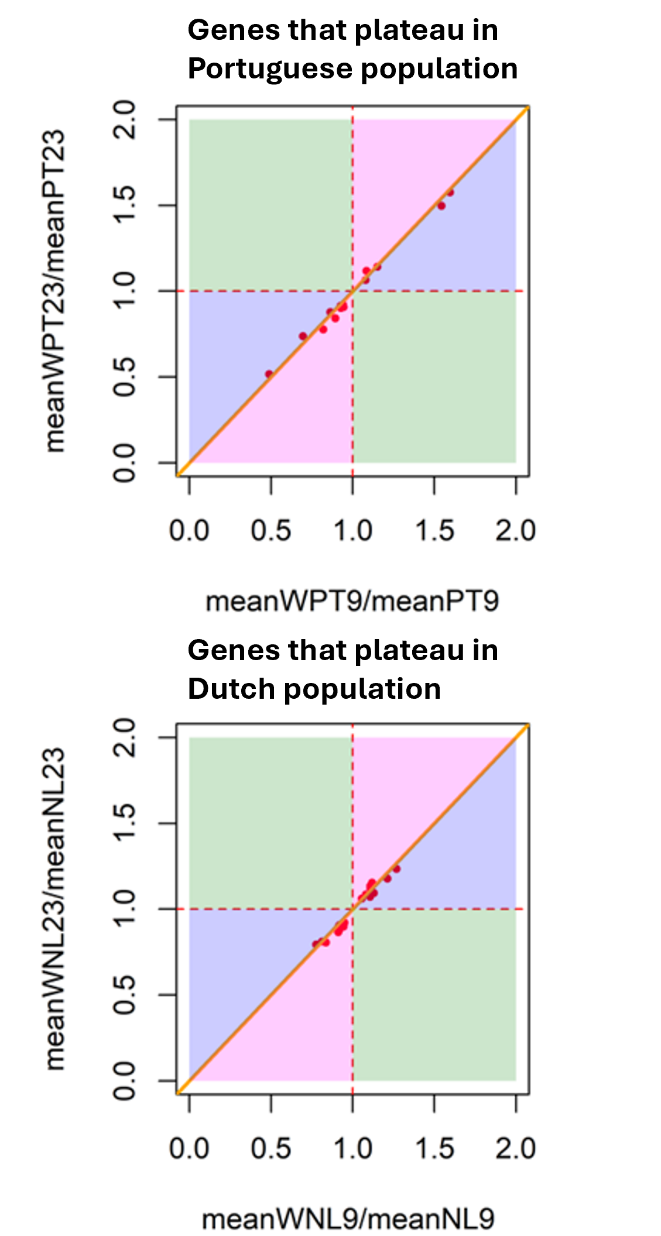


Figure S2 - Scatter plots for the genes that present a plateauing pattern. Portuguese populations are shown in the upper panel and Dutch in the lower panel. The x-axis refers to the ratio of expression between warming and control populations in the 9th generation (e. g. WPT9/PT9) and the y-axis represents the same ratio in the 23rd generation (e.g., WPT23/PT23). Red dots are candidate genes in common between generations. All the dots are colored red since being a candidate gene in both generations was a condition to be considered a gene plateauing.


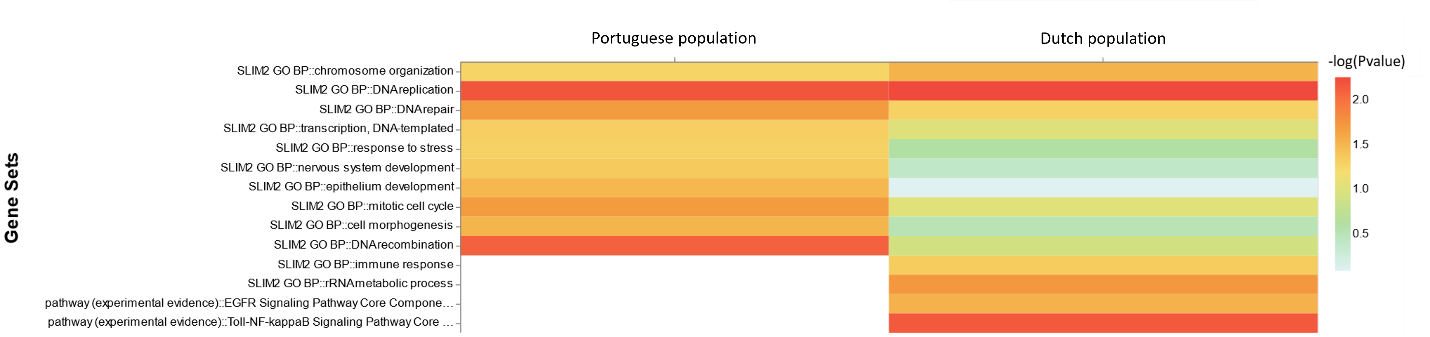


Figure S3 - Heat Map of functional characterization of genes with reversal patterns across generations. The first and second columns refer to genes from Portuguese and Dutch populations respectively. The color gradient indicates different magnitudes of -log(p-value).
